# Applications of Nanomaterials in Restorative Dentistry and Endodontics: A Narrative Review

**DOI:** 10.3390/ma19132786

**Published:** 2026-07-01

**Authors:** Marina A. Marciano, Jennifer S. Pereira, Thiago B. M. Antunes, Paulo J. Palma

**Affiliations:** 1 Department of Restorative Dentistry, University of Campinas (UNICAMP), Piracicaba 13414-903, SP, Brazil; marinama@unicamp.br (M.A.M.); jenniferspodonto@gmail.com (J.S.P.); thiagobessa1999@gmail.com (T.B.M.A.); 2 Faculty of Medicine, Center for Innovation and Research in Oral Sciences (CIROS), Institute of Endodontics, University of Coimbra, 3000-075 Coimbra, Portugal

**Keywords:** nanotechnology, nanoparticles, restorative dentistry, endodontics, biomaterials, remineralization

## Abstract

Nanotechnology has emerged as a promising strategy in restorative dentistry and endodontics due to the physicochemical and biological properties of nanomaterials. This narrative review aimed to critically analyze the current applications of nanomaterials in restorative dentistry and endodontics, highlighting their mechanisms of action, biological properties, and translational potential. A literature search was performed in the PubMed/MEDLINE database using combinations of MeSH terms and free keywords related to nanomaterials and dental applications. Studies published in English within the last twenty years and addressing restorative or endodontic applications were considered. After screening and eligibility assessment, 69 studies were included in the descriptive analysis. The findings indicate that nanomaterials have been investigated in preventive strategies, adhesive systems, restorative materials, intracanal medicaments, endodontic sealers, vital pulp therapy, and regenerative formulations. In restorative dentistry, nanoparticles such as silver nanoparticles, nano-hydroxyapatite, amorphous calcium phosphate, bioactive glass nanoparticles, and chitosan-based systems showed favorable antimicrobial, remineralizing, and material-enhancing properties. In endodontics, silver and chitosan nanoparticles showed potential for intracanal disinfection and biofilm disruption, while chlorhexidine, zinc, and bioactive glass nanoparticles enhanced the antimicrobial activity and sealing ability of endodontic sealers. In addition, magnetic nanoparticles, mesoporous silica nanoparticles, and hydroxyapatite nanoparticles presented promising applications in regenerative endodontics and vital pulp therapy. However, most of the available evidence is still based on in vitro studies, with limited long-term clinical validation. Overall, nanotechnology offers potential experimental advantages for improving preventive, restorative, and endodontic therapies; however, its successful clinical translation remains strictly dependent on overcoming critical biosafety barriers and addressing long-term toxicity concerns.

## 1. Introduction

Dental caries is a multifactorial and dynamic disease resulting from the interaction among dental biofilm, dietary sugars, and the continuous cycles of demineralization and remineralization occurring at the tooth surface [1]. It affects individuals at all stages of life and may involve both primary and permanent dentitions, compromising the crown and, particularly in adults, exposed root surfaces [2]. The susceptibility of dental tissues to caries is closely associated with their mineral composition, predominantly formed by hydroxyapatite crystals [3]. Under acidic conditions generated by bacterial metabolism within the biofilm, these crystals undergo dissolution, leading to mineral loss [4]. 

In addition to cariogenic acids, erosive acids may further contribute to the degradation of dental hard tissues [5]. As enamel is continuously exposed to chemical and mechanical challenges, the underlying dentin may become exposed, resulting in enlargement of dentinal tubules and increased dentin permeability [6,7]. When acidic challenges occur frequently or persist over time, mineral loss exceeds the natural remineralization capacity mediated by saliva and fluoride, leading to progressive enamel breakdown and subsequent dentin involvement [8]. This process compromises tooth structural integrity and often requires restorative intervention to recover lost tissue, function, and esthetics.

Furthermore, pH fluctuations in the oral cavity play a critical role that extends beyond cariogenic demineralization, affecting both tissue integrity and the longevity of dental treatments [3]. In addition to promoting dental erosion and the enlargement of dentinal tubules—processes that lead to hypersensitivity and increased dentin permeability—acidic environments facilitate the chemical and physical degradation of restorative materials and adhesives [9]. This degradation often favors marginal microleakage and potential reinfection of the pulp-dentin complex [10]. From a biosafety perspective, pH variations are also critical to the stability of nanomaterials, as they may induce the unintended release of metallic ions and nanoparticles into the oral environment, raising concerns regarding cellular and systemic toxicity [10].

Among the restorative materials currently available, composite resins are widely used because of their favorable esthetic characteristics [11], satisfactory mechanical performance, and ability to bond to dental substrates through adhesive systems [12]. Nevertheless, despite substantial advances in material formulations and adhesive strategies, marginal microleakage and the development of secondary caries remain among the leading causes of restoration failure and replacement [13].

Secondary caries commonly develops at the tooth–restoration interface, where marginal gaps, inadequate adaptation, and biofilm accumulation facilitate bacterial colonization and acid production, promoting new demineralization events [13]. Furthermore, as carious lesions progress beyond enamel and reach dentin, the increased permeability of the substrate and the diffusion of bacterial byproducts toward the pulp may trigger inflammatory responses that can ultimately culminate in pulp necrosis if left untreated [14]. Under these conditions, restorative procedures alone may become insufficient, requiring endodontic intervention to eliminate infection, disinfect the root canal system, and preserve the affected tooth [15].

In this context, nanotechnology has emerged as an interesting strategy in restorative dentistry and endodontics [16,17]. Nanomaterials and nanoparticles have been extensively investigated because of their potential applications across different stages of dental treatment, ranging from prevention and remineralization to restorative and endodontic therapies, including regenerative approaches [18,19,20].

Nanomaterials comprise a broad category of materials defined by regulatory organizations such as the European Commission and the Food and Drug Administration (FDA) as materials presenting at least one dimension within the nanoscale range of 1–100 nm or exhibiting properties characteristic of this scale, including films, fibers, aggregates, and particles [21]. Nanoparticles, in turn, represent a specific subgroup of nano-objects in which all three dimensions are below 100 nm, according to ISO definitions [22]. The reduced size of nanoparticles provides a high surface area-to-volume ratio, significantly enhancing their chemical reactivity, interaction with biological systems, and functional performance [16]. These characteristics enable the development of restorative and endodontic materials with improved mechanical properties, enhanced antimicrobial activity, greater structural stability, bioactivity, and superior integration with dental tissues [23].

Therefore, understanding current nanotechnologies and their applications in restorative dentistry and endodontics may contribute to improving clinical outcomes and therapeutic performance. Accordingly, the aim of this review was to critically evaluate the current applications of nanomaterials in restorative dentistry and endodontics, emphasizing their mechanisms of action, biological properties, clinical relevance, and translational potential.

## 2. Methods

The present study discusses nanomaterial applications in restorative dentistry and endodontics. It focuses on their preventive, antimicrobial, bioactive, remineralizing, and regenerative properties. The literature search was performed in the PubMed/MEDLINE database using combinations of MeSH terms and free keywords related to nanomaterials and dental applications. To improve search specificity and organization, the strategy was divided into two thematic domains: restorative dentistry and endodontics. The terms were combined using the Boolean operators AND and OR.

- For restorative dentistry, the following search strategy was applied: (“nanomaterials” OR “nanoparticles”) AND (“restorative dentistry” OR “dental adhesives” OR “remineralization” OR “biofilms”).
- For endodontics, the search strategy included: (“nanomaterials” OR “nanoparticles”) AND (“endodontics” OR “root canal therapy” OR “sealers” OR “irrigants” OR “regenerative endodontics”).

Studies published in English between 2006 and 2 June 2026 were prioritized. The selection of studies was performed independently by two reviewers (J.S.P. and T.B.M.A) in two consecutive stages: title/abstract screening and full-text assessment. Any disagreements were resolved through discussion between the reviewers.

Relevant in vitro, in vivo, and clinical studies addressing restorative and endodontic applications were included. Exclusion criteria comprised literature reviews, duplicate articles, and conference abstracts. We also excluded editorials, comments, study protocols, and book chapters.

Data related to the general physicochemical properties and biological activity of the nanomaterials were extracted from the included studies. Subsequently, the selected articles were descriptively analyzed and organized according to their main clinical and functional applications, including preventive strategies, adhesive systems, restorative materials, intracanal disinfection, endodontic sealers, and regenerative endodontic approaches.

Due to the narrative nature of this review, no formal risk-of-bias assessment or quantitative meta-analysis was performed. The aim was to provide a comprehensive and critical overview of the current state of nanotechnology applications in restorative dentistry and endodontics, as well as to discuss future perspectives and translational challenges.

## 3. Results

**Figure 1:**
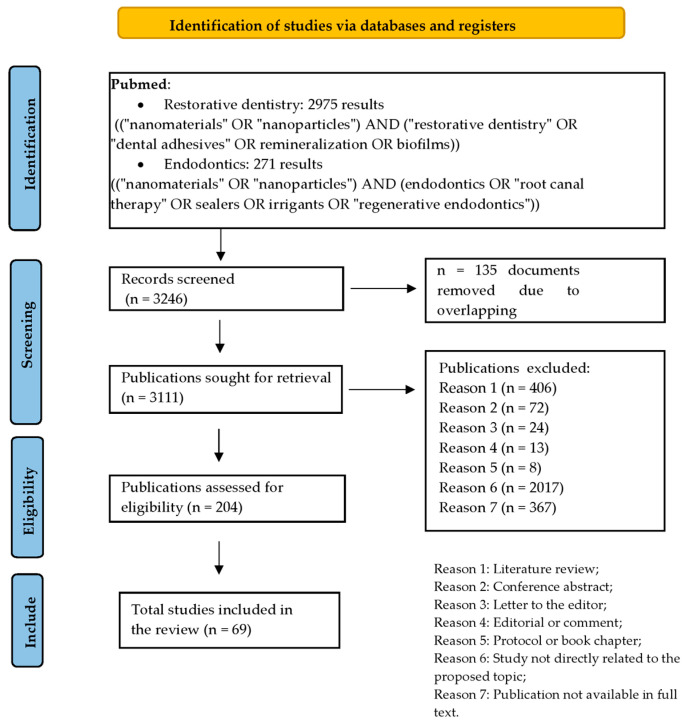
Flowchart illustrating the study identification, screening, eligibility assessment, and inclusion process for the current study of nanomaterial applications in restorative dentistry and endodontics, based on searches performed in the PubMed/MEDLINE database.

The study selection process is illustrated in the PRISMA flow diagram (Figure 1). The electronic search conducted in the PubMed database identified a total of 3246 records, including 2975 studies related to restorative dentistry and 271 related to endodontics. After the removal of 135 duplicate records, 3111 publications were screened by title and abstract. Subsequently, 204 full-text articles were assessed for eligibility.

During the eligibility assessment, studies were excluded for the following reasons: literature reviews (*n* = 406), conference abstracts (*n* = 72), letters to the editor (*n* = 24), editorial comments (*n* = 13), protocols or book chapters (*n* = 8), studies not directly related to the scope of the present review (*n* = 2017), and publications without full-text availability (*n* = 367). After the systematic application of the inclusion and exclusion criteria, a total of 69 studies were considered eligible and included in this present review.

The selected studies were subsequently organized into seven major categories according to their primary applications in restorative dentistry (Table 1) and endodontics (Table 2):

1. Preventive strategies (*n* = 14) focused on remineralization, biofilm modulation, hypersensitivity reduction, and enamel protection through systems such as CPP-ACP nanocomplexes, nano-hydroxyapatite, and silver nanoparticles.
2. Adhesive systems (*n* = 12) primarily investigated antibacterial activity, matrix metalloproteinase (MMP) inhibition, dentin remineralization, and bond durability.
3. Functional nanoparticles in restorative materials (*n* = 15) addressed antimicrobial activity, mechanical reinforcement, biofilm inhibition, and restoration durability.
4. Auxiliary function nanoparticles (*n* = 4) included applications for antimicrobial enhancement, biomimetic enamel regeneration, and the improvement of biological and mechanical properties of restorative materials.
5. Endodontic irrigants and intracanal medicaments (*n* = 6) targeted antimicrobial activity, dentinal tubule disinfection, and biofilm disruption.
6. Endodontic sealers and filling materials (*n* = 10) focused on antimicrobial reinforcement, controlled drug release, bioactivity, and sealing improvement.
7. Regenerative endodontic formulations (*n* = 8) involved nanoparticle systems designed to support pulp regeneration, odontogenic differentiation, and revascularization procedures.

Most of the included studies consisted of in vitro investigations, reflecting the predominantly experimental nature of nanotechnology research in restorative dentistry and endodontics. Additional study designs included in vivo experimental studies, randomized controlled clinical trials, prospective clinical studies, and in situ investigations.

## 4. Nanomaterial Synthesis Methods

Research in nanotechnology has advanced significantly due to the unique properties exhibited by materials at the nanoscale [88]. These features may confer unique properties and enhanced performance compared with conventional materials, thereby promoting their application in different fields of dentistry [89]. The incorporation of nanomaterials into dental biomaterials has shown the potential to enhance stability, mechanical strength, and bioactivity, potentially improving laboratory performance. These enhancements may eventually contribute to a more favorable cost–benefit relationship, although long-term clinical validation is still required.

The synthesis of nanomaterials enables the development of structures with specific physicochemical and biological properties, which are strongly influenced by parameters such as particle size, shape, morphology, and distribution [90]. Since these characteristics are directly related to the synthesis route employed, nanomaterial production methods are generally classified into two main approaches: top-down and bottom-up [91].

The top-down approach consists of reducing macroscopic materials into nanometric structures through physical processes, such as milling, grinding, or lithography [92]. In contrast, the bottom-up approach is based on the controlled organization of atoms, molecules, or clusters to form nanostructures [92]. Several techniques have been described for nanomaterial synthesis, including chemical reduction [93,94], electrochemical reduction [95], photochemical reactions in reverse micelles [96], and green chemistry-based methods [97].

In addition, specific synthesis methods, such as sol–gel, microemulsion, and flame spray, have gained considerable attention in the development of bioactive nanoparticles due to their direct influence on properties such as surface area, porosity, chemical homogeneity, and bioactivity [98]. The sol–gel method allows high structural control and chemical purity, whereas microemulsion techniques favor lower particle aggregation and greater particle uniformity [98]. Flame spray synthesis, in turn, stands out for its high productivity and the production of highly bioactive amorphous structures. Nevertheless, each technique presents limitations related to cost, yield, operational complexity, and processing conditions, indicating that the choice of synthesis method directly influences the biological performance and clinical applicability of nanomaterials [98].

## 5. Fundamentals of Nanotechnology in Dentistry

### 5.1. Classification of Nanomaterials and Nanoparticles

Nanomaterials are defined as materials presenting at least one dimension within the nanometric scale and may assume different structural forms, such as nanoparticles, nanotubes, nanofibers, nanocrystals, nanofilms, and nanospheres [91,99,100]. Nanoparticles, in turn, represent a specific class of nanomaterials characterized by nanometric dimensions in all directions [101].

Nanomaterials can be classified according to their dimensionality [21]:

Zero-dimensional (0D) nanomaterials, in which all dimensions are confined to the nanoscale, are typically represented by nanoparticles with a nearly spherical morphology. Common examples in dentistry include silver nanoparticles (AgNPs) [102,103], zinc oxide (ZnO) [104], and nanohydroxyapatite (nHAp) [105]. Their high surface area-to-volume ratio enhances chemical reactivity [106], favoring rapid ion release—such as calcium and phosphate ions involved in remineralization—as well as interactions with bacterial membranes, contributing to their antimicrobial activity.

One-dimensional (1D) nanomaterials exhibit an elongated structure, with one dimension extending beyond the nanoscale, as seen in nanotubes and nanowires [107]. This geometry enables a more directed functional behavior along their length. For instance, aluminosilicate nanotubes (AlNTs) [108] have been explored as carriers for the sustained release of therapeutic agents and for dentinal tubule occlusion. Similarly, self-assembling peptides can organize into nanofibrous scaffolds that act as templates for hydroxyapatite nucleation, supporting biomimetic mineralization.

Two-dimensional (2D) nanomaterials are characterized by a lamellar structure, in which thickness remains at the nanoscale while the other dimensions are extended. In dental applications, materials such as nano-fluorapatite and carbonated nanohydroxyapatite can form continuous, mineral-like coatings on tooth surfaces. This configuration is particularly advantageous for creating protective interfaces, as it supports protein adsorption and guides the organized deposition of mineral phases on enamel and dentin [103].

Nanoparticles (NPs), in turn, can be classified based on different criteria, including their origin, chemical composition, and structural configuration. Regarding origin, they may be natural, such as silver and copper, or synthetic, such as graphene and chitosan [109]. In terms of composition, nanoparticles can be organic, such as dendrimers and ferritin; inorganic, such as carbonates, carbides, phosphates, and metal oxides; or carbon-based, such as fullerenes, carbon black, and carbon quantum dots [110].

From a structural perspective, they include carbon-based nanoparticles (e.g., fullerenes, graphene, and amorphous carbon), metallic nanoparticles (e.g., gold, silver, and metal oxides, including titanium oxide, zinc oxide, and copper oxide) [111], as well as more complex structures such as dendrimers, liposomes, and ferritin; This structural diversity is associated with their potential for application in both restorative dentistry and endodontics, contributing to the enhancement of material properties and the optimization of clinical outcomes [109,110,112]. As nanoparticles can enhance key material properties such as mechanical strength, chemical stability, surface polishability, esthetics, and antimicrobial activity, ultimately contributing to potentially improve the laboratory performance of restorations, although clinical confirmation is still pending [113].

### 5.2. Applications of Nanoparticles in Restorative Dentistry

Nanoparticles have been investigated for applications at different stages of restorative dentistry, from minimally invasive approaches to definitive restorative procedures. They may be incorporated into sealants [114], protective coatings [115], and remineralizing materials [116], contributing to the prevention of demineralization, biofilm control, and reduction of dentin hypersensitivity [33,40].

Nanoparticles can also be incorporated into etchants [117], primers, and adhesive systems [47] to improve antimicrobial and adhesive properties. These effects may lead to greater stability at the tooth-restoration interface. In composite resins and restorative biomaterials, nanoparticles may also improve mechanical strength, chemical stability, polishability, and esthetic properties, thereby contributing to greater restorative durability [16].

Certain nanoparticles also exhibit multifunctional properties, such as silver nanoparticles (AgNPs) [57], zinc oxide (ZnO) [118], and nano-hydroxyapatite (nHAp) [33], since their high reactivity and elevated surface area-to-volume ratio enable simultaneous action in tissue remineralization, biofilm control, and reinforcement of adhesive interfaces [119]. This characteristic favors the development of synergistic therapeutic strategies, in which different properties—such as antimicrobial activity, mechanical reinforcement, and remineralizing potential—act in an integrated manner to increase the longevity of restorative treatments [31,116,120,121]. Table 1 summarizes the main nanoparticles used in restorative dentistry, emphasizing their functional classification and respective clinical applications.

### 5.3. Nanomaterials Applied to Prevention and Remineralization

Secondary caries remains a significant challenge in restorative dentistry due to its detrimental effects on restoration longevity and treatment costs [122]. Occurring adjacent to existing restorations, it is one of the leading causes of restorative failure. Its management often requires repeated restoration replacement, resulting in progressive loss of healthy dental tissue and weakening of tooth structure [13]. Despite advances in restorative materials and preventive strategies, effective clinical control of secondary caries remains difficult because of its multifactorial etiology [23].

Nanotechnology-based preventive strategies have attracted considerable interest for promoting remineralization and limiting the progression of early dental lesions [123]. Available evidence, predominantly derived from laboratory studies, indicates promising effects on remineralization and biofilm control. Nevertheless, the predominance of in vitro, in situ, and short-term investigations restricts the translation of these findings into clinical practice.

Casein phosphopeptide–amorphous calcium phosphate (CPP-ACP) nanocomplexes have been proposed as a bioactive approach to enhance enamel remineralization by serving as reservoirs of calcium (Ca^2+^) and phosphate (PO_4_^3−^) ions within the dental biofilm [24]. Although preclinical studies have reported promising results, evidence supporting their superiority over conventional fluoride therapies remains limited. A randomized controlled trial found that sodium fluoride (NaF) varnishes containing CPP-ACP were no more effective than conventional NaF varnishes in preventing carious lesions in high-risk children [25], indicating that the added benefit of CPP-ACP supplementation remains uncertain.

In contrast, formulations containing silver nanoparticles combined with xylitol were more effective in reducing white spot lesions than mouthwashes containing 0.05% chlorhexidine or fluoride alone [124]. These findings suggest that nanoparticle-based formulations may offer additional benefits for caries prevention.

Daily-use dentifrices containing nano-calcium carbonate may act as a sustained source of calcium ions following rinsing, enhancing calcium availability in the oral environment and promoting enamel remineralization [27]. However, their effectiveness appears to depend on factors such as nanoparticle concentration, formulation stability, and frequency of use.

Calcium glycerophosphate has attracted interest as a preventive agent because of its buffering capacity and its ability to increase calcium and phosphate concentrations within the dental biofilm [28,29]. Studies have also reported reductions in microbial biomass and favorable interactions with dental tissues, supporting its use in dentifrice formulations [30,31]. However, the mechanisms underlying these effects have not yet been fully elucidated.

In xerostomia-associated conditions, the risk of caries development is increased. In this context, silver nanoparticles (AgNPs) have received attention because of their broad antimicrobial activity [32]. Their antimicrobial effects are attributed to mechanisms such as bacterial membrane disruption, interference with enzymatic processes, and inhibition of biofilm formation, particularly against *Streptococcus mutans*. Despite promising laboratory evidence, variations in particle size, concentration, and delivery systems hinder direct comparisons among studies and limit the development of standardized therapeutic protocols [125].

Nano-hydroxyapatite (nHAp) has been extensively investigated due to its chemical similarity to dental enamel [126]. Dentifrices containing nHAp have been shown to promote the remineralization of early enamel lesions, reduce acid-induced demineralization, and alleviate dentin hypersensitivity [33]. Improvements in surface smoothness and tooth gloss have also been reported [34]. However, current clinical evidence remains heterogeneous, particularly regarding optimal particle concentration, long-term stability, and efficacy in primary teeth [126].

Modified formulations, such as nanofluorapatite (nFA), have been proposed to combine the remineralizing effects of hydroxyapatite with sustained fluoride release. Studies suggest that dentifrices, varnishes, and restorative materials containing nFA may increase enamel resistance under acidic conditions through the formation of fluoride-rich mineral layers [36,37]. In addition, their interaction with dentin may promote dentinal tubule occlusion and reduce hypersensitivity [35]. Despite these favorable findings, the long-term stability of the deposited mineral layer has not yet been fully established.

Therefore, while favorable results have been observed in several in situ and laboratory models, the routine clinical applicability of these nanotechnology-based preventive strategies is not yet supported by robust clinical evidence, and their definitive superiority over conventional therapies remains inconclusive.

### 5.4. Nanoparticles in Adhesive Systems

The heterogeneous structure of dentin, together with its high organic content and the susceptibility of adhesive systems to hydrolytic degradation, compromises the long-term stability of the resin–dentin interface [127]. To address these challenges, various nanoparticle-based approaches have been explored to enhance the antimicrobial, bioactive, and mechanical properties of this interface. However, most of the available evidence is still derived from laboratory studies.

Silver nanoparticles (AgNPs) exhibit antimicrobial activity through bacterial membrane disruption and interactions with cellular components [38,81], demonstrating promising effects on biofilm control in experimental models. Similarly, nanoparticulate amorphous calcium phosphate (NACP) has been investigated for its ion-releasing capacity and remineralizing potential, which may contribute to adhesive interface stability [40,42,116].

Bioactive glass nanoparticles (BGNs) and nano-hydroxyapatite (nHAp) have demonstrated the ability to promote mineral deposition and occlude demineralized dentin areas in vitro [43]. Similarly, zinc (ZnNPs) and gold nanoparticles (AuNPs) may contribute to collagen matrix preservation through the inhibition of matrix metalloproteinases (MMPs) under experimental conditions [44,45].

Controlled-release systems, including mesoporous silica nanoparticles (MSNs) and aluminosilicate nanotubes (AlNTs), have been explored as platforms for the sustained delivery of therapeutic agents while maintaining mechanical properties [128]. Reactive nanogels (RNGs) may further enhance polymer network integrity and reduce water permeability [129].

Copper nanoparticles and poly(acrylic acid)-coated copper iodide particles have shown sustained antimicrobial activity in vitro and may contribute to preserving adhesive interface integrity [61,130]. Likewise, β-tricalcium phosphate nanoparticles may promote mineral deposition and dentinal tubule occlusion, depending on their physicochemical characteristics [49]. Fluoride-functionalized formulations have also been associated with reduced dentin erosion in laboratory models [6].

Titanium dioxide nanoparticles (TiO_2_-NPs) have been investigated for their reinforcing, photocatalytic, and antimicrobial properties [131,132]. In glass ionomer cements, they have been associated with enhanced mechanical strength and antibacterial activity against *Streptococcus mutans* [133,134], although these effects are influenced by particle concentration and dispersion.

Superparamagnetic iron oxide nanoparticles (SPIONs) have attracted interest for their ability to enhance penetration into acid-etched dentin and improve bond strength in experimental models [62]. They also exhibit antimicrobial activity under laboratory conditions.

Gold nanoparticles (AuNPs) have been investigated as additives in adhesive systems and resin composites because of their potential to enhance mechanical properties and polymer stability [19]. In vitro studies have demonstrated improvements in bond strength and microhardness, along with potential antimicrobial activity [64,135].

Overall, although nanoparticles confer multifunctional potential to adhesive systems, the available evidence remains predominantly in vitro, limiting direct clinical extrapolation. While in vitro improvements in immediate bond strength and adhesive performance have been reported under laboratory conditions, the long-term stability of the resin–dentin interface remains uncertain due to the limited number of studies evaluating aging conditions such as thermocycling and prolonged water storage.

### 5.5. Functional Nanoparticles for Restorative Materials

Although composite resins remain widely used in clinical practice due to their favorable esthetic and adhesive properties [76,136], their greater susceptibility to biofilm accumulation compared with materials such as amalgam and glass ionomer still represents an important limitation [137,138]. In this context, experimental strategies involving the incorporation of nanoparticles into restorative materials aim to address failures associated with marginal leakage and recurrent caries; however, routine clinical applicability remains unproven.

Among the nanoparticle-based approaches, antimicrobial nanoparticles capable of interfering with bacterial adhesion, cell membrane integrity, and microbial metabolic pathways have attracted considerable attention. Quaternized polyethyleneimine nanoparticles, for example, demonstrate contact-dependent antimicrobial activity and the potential to reduce bacterial recolonization [50,51]. Similarly, multifunctional systems containing chlorhexidine and reactive calcium phosphate associated with silica–silicon carbide nanoparticles simultaneously exhibit antibacterial, adhesive, and remineralizing properties [52].

Metallic nanoparticles [139], such as silver, zinc, copper, titanium, iron, and gold nanoparticles, have been widely employed due to the combination of mechanical, chemical, and optical properties that contribute to improving the performance and stability of restorative materials [19,140,141].

Hybrid zinc oxide–silver and copper oxide–silver nanoparticles promote increased permeability and disruption of the bacterial membrane in *B. subtilis*, *S. aureus*, and other species [54], whereas copper-doped titanium dioxide nanoparticles reduce enzymatic activity and alter metabolic pathways in *M. smegmatis* [53]. Silver nanoparticles act through adsorption and cellular penetration, with toxicity mediated by electrostatic interactions in *K. pneumoniae* [56], while zinc oxide nanoparticles generate reactive oxygen species (ROS) and promote membrane disruption in *P. aeruginosa* [58].

Other metallic oxides, such as aluminum oxide and nickel oxide, compromise structural integrity and bacterial adhesion in *Escherichia coli* [59,142], whereas titanium dioxide may induce mutations through oxidative stress in *S. typhimurium* [60]. Similarly, metallic silver interferes with permeability, cell division, and sulfur- and phosphorus-containing components in *E. coli* [102], highlighting the broad antimicrobial versatility of these nanomaterials.

Silver nanoparticles (AgNPs) have been widely investigated because of their antimicrobial activity and low cytotoxicity at appropriate concentrations [57]. Their incorporation into composites, adhesives, and primers may inhibit microbial growth without compromising mechanical or esthetic properties [140,143].

Zinc oxide (ZnO) and titanium dioxide (TiO_2_) nanoparticles may reduce biofilm formation and enhance wear resistance and compressive strength [144,145]. However, high concentrations can lead to nanoparticle agglomeration, compromising optical properties, adhesive stability, and mechanical performance. Moreover, the antimicrobial activity of TiO_2_ depends on light activation, which may not be consistently available in the oral environment.

More recent approaches include superparamagnetic iron oxide nanoparticles (SPIONs), which may enhance adhesive infiltration and bond strength through magnetic field application [62]. However, their long-term stability remains unclear. Gold nanoparticles (AuNPs) have been investigated for their reinforcing, stabilizing, and antibacterial properties [19]. Studies have reported increased bond strength and microhardness without compromising optical properties [64,146], although their high production cost may limit clinical application.

### 5.6. Auxiliary Function Nanoparticles

Chitosan nanoparticles can be used as a versatile additive in restorative dentistry, acting from prevention to material modification [147]. Chitosan-based dentifrices have been shown to significantly reduce tissue loss even in the absence of fluoride, indicating their protective potential against wear and demineralization [65]. In enamel regeneration, chitosan hydrogels have been used as efficient vehicles for the delivery of amelogenin in superficial defects, favoring the reorganization of the enamel crystalline structure [66]. At the adhesive interface, chitosan is explored as a bioadhesive matrix capable of improving the integrity of the hybrid layer; antioxidant chitosan hydrogels associated with propolis, nystatin, or β-carotene have been shown to increase shear bond strength and reduce nanoleakage [67,148]. In addition, its use as a nano-additive in glass ionomer cements contributes to mechanical and biological enhancements, strengthening the bond to the dental substrate and expanding the clinical performance of these materials [68].

## 6. From Caries to Endodontic Therapy: A Nanotechnology-Based Continuum

Dental caries is a progressive demineralization process that may gradually affect dentin and eventually reach the dental pulp. In early stages, biofilm control and remineralization strategies aim to prevent lesion progression and preserve dental structure [2,149]. However, when pulp exposure occurs, conservative procedures such as direct pulp capping and pulpotomy may be indicated to maintain pulp vitality and function [84,150]. In more advanced conditions, characterized by irreversible inflammation or pulp necrosis, endodontic treatment becomes necessary to disinfect the root canal system and promote periapical repair [151].

## 7. Functional Classification of Nanoparticles in Endodontics

Nanoparticles in endodontics can be organized according to their main clinical function, as summarized in Table 2, which presents the main nanoparticles and their respective clinical applications [152].

A first category includes nanoparticles incorporated into irrigating solutions and intracanal medicaments, aiming to enhance antimicrobial activity and promote greater disruption of biofilm within the root canal system, particularly in areas that are difficult to access [153]. A second group comprises nanoparticles integrated into endodontic sealers and filling materials, contributing to improved physicochemical properties, enhanced sealing ability, and increased bioactivity, all of which are essential for the success of endodontic treatment [154].

A third group includes nanoparticles that can be incorporated into regenerative formulations to enhance their biological performance, improving interaction with periapical tissues and supporting tissue repair processes [108,155,156,157].

Although this functional classification facilitates the understanding of their clinical applications, it is important to recognize that many nanomaterials exhibit multifunctional behavior, extending beyond rigid categories. Nanoparticles such as silver (AgNPs), zinc oxide (ZnO), and nano-hydroxyapatite (nHAp) exemplify this versatility, as they can simultaneously exert antimicrobial effects, enhance material performance, and interact with periapical tissues, contributing to more integrated and effective therapeutic strategies.

### 7.1. Nanotechnology in Vital Pulp Therapy

Although calcium hydroxide remains widely used for direct pulp capping because of its antibacterial activity and mineralization-inducing properties [158], its long-term clinical performance remains questionable. In a study of 401 teeth with carious pulp exposures, direct pulp capping with calcium hydroxide showed success rates of 37% at 5 years and only 13% at 10 years [159].

Nanostructured systems show promise for dentin-pulp regeneration, although biological predictability and protocol standardization remain major challenges [160]. Hydrogels containing cerium oxide nanoparticles associated with DMP1 demonstrated modulation of inflammation and odontogenic differentiation of hDPSCs [82]. Similarly, nano-HAP systems combined with FGF-2 promoted neovascularization and mineralized tissue formation through sustained release of bioactive factors [100]. However, differences in nanoparticle composition and experimental protocols still limit direct comparisons among studies [160].

In primary and immature permanent teeth, pulpotomy aims not only to preserve pulp vitality but also to maintain root development [161]. In these cases, the procedure also aims to maintain the cellular activity required for physiological root development, favoring the formation and maturation of dental structures [162]. A clinical study evaluating magnetic nanoparticles (MNPs) in pulpotomy of primary molars reported a 98% clinical success rate after 1 year, with favorable radiographic and histological findings, including odontoblastic proliferation and preservation of viable pulp tissue [84].

Mesoporous bioactive glass nanoparticles (MBGNs) have also been proposed for dentin-pulp regeneration. These systems may enhance ion release, stimulate apatite formation, and provide antimicrobial and biocompatible effects that help maintain a favorable pulpal microenvironment during the healing process [85].

### 7.2. Nanoparticle-Based Strategies for Root Canal Disinfection

These nanoparticles enhance disinfection through specific mechanisms of action. Their physicochemical properties improve diffusion and biofilm interaction compared to conventional methods [163]. This enhanced performance is supported by their ultrasmall size and high surface area-to-volume ratio [164], which confer increased chemical reactivity and facilitate diffusion and penetration into complex anatomical niches of the root canal system, including dentinal tubules and irregularities of the canal walls [165].

One representative example is silver nanoparticles (AgNPs), which act through multiple mechanisms, including disruption of bacterial cell membranes, generation of reactive oxygen species, and interference with DNA replication, resulting in potent antimicrobial activity [143]. However, due to the requirement for prolonged contact time to achieve maximum efficacy, their application is more suitable as an intracanal medicament rather than as a standalone irrigant [166].

The association of AgNPs with calcium hydroxide [Ca(OH)_2_] demonstrated greater reduction of *Enterococcus faecalis* colony-forming units after one week compared with Ca(OH)_2_ alone [69]. In addition, AgNP-containing gel formulations may improve material retention within the root canal system and prolong interaction with bacterial cell walls, enhancing antimicrobial activity [167].

However, despite their therapeutic potential, recent studies suggest that prolonged exposure to AgNPs may induce adaptive mechanisms in endodontic microorganisms [168]. Reported mechanisms include changes in membrane permeability, increased efflux pump expression, reduced porin expression, and plasmid-mediated resistance gene acquisition [168]. In addition, subinhibitory concentrations may favor the survival of persistent microorganisms such as *E. faecalis* [166]. These findings indicate that the clinical use of AgNPs still requires caution regarding concentration, exposure time, and the risk of microbial adaptation.

Chitosan nanoparticles (CSNPs) have also gained attention because their positive charge enhances interaction with biofilms and bacterial cell walls [147]. In addition, they may function as controlled drug delivery systems. In an in vitro root canal model, chitosan paste promoted greater reduction of viable *E. faecalis* biofilm and *Candida albicans* after 7 days compared with Ca(OH)_2_ [70].

The combination of cerium nitrate and dextran shows potential to induce biomimetic mineralization associated with antibacterial activity, as it promotes the formation of a mineral barrier capable of occluding dentinal tubules and lateral ramifications of the root canal system [169]. This effect contributes to the reduction of microbial colonization niches and, consequently, to the stability of the seal and the success of endodontic treatment [169].

Another study evaluating an intracanal medicament based on Jasminum, reinforced with titanium dioxide nanoparticles and calcium hydroxide (Ca(OH)_2_), compared with conventional Ca(OH)_2_ in patients with symptomatic apical periodontitis, showed a significant reduction in postoperative pain. This effect was characterized by a decrease in pain intensity as early as 4 h after treatment, with sustained analgesic control for up to 96 h [72]. These findings suggest that the incorporation of nanoparticles may enhance the biological performance of Ca(OH)_2_, promoting faster and more prolonged postoperative pain control [72]. Although these findings are relevant, it is important to consider that clinical studies involving nanoparticles remain limited in number and frequently present short follow-up periods.

The use of lasers as adjuncts in root canal system disinfection has demonstrated high penetration capacity into dentinal tubules (>400 µm) and strong bactericidal efficacy, reaching reduction rates close to 99%, in addition to promoting dentinal tubule occlusion [170,171]. Laser activation of antimicrobial agents, such as nanoparticles (AgNPs, chitosan) and chlorhexidine, has generally shown greater effectiveness compared to conventional irrigation techniques, including side-vented needles and ultrasonic agitation [74,172,173,174,175].

However, substantial heterogeneity among studies regarding laser parameters, such as wavelength, power settings, irradiation mode, fiber diameter, and activation time, limits direct comparisons and prevents protocol standardization [74,172,174]. Variations in nanoparticle concentration, exposure time, and biofilm models also contribute to inconsistent findings. Most studies use simplified models, including planktonic cultures or monospecies Enterococcus faecalis biofilms, which may not accurately reproduce clinical conditions. Although laser activation alone may be insufficient for complete disinfection [173,176], its association with nanoparticles significantly enhances Enterococcus faecalis reduction, suggesting a synergistic effect [174].

Phototherapy mediated by polydopamine nanoparticles (PDA) activated by near-infrared light (808 nm) has shown antimicrobial effects against *Enterococcus faecalis* [73], consistent with laser-activated disinfection strategies. This effect results from NIR-to-heat conversion, leading to bacterial membrane damage and biofilm disruption [177]. Although the PDA + NIR system demonstrated higher bactericidal rates than 2.5% NaOCl in vitro, these findings should be interpreted cautiously, as they may not reflect clinical superiority under routine endodontic conditions [73].

### 7.3. Nanoparticles in Endodontic Sealers and Filling Materials

The incorporation of nanoparticles into endodontic sealers may enhance biological and physicochemical properties, particularly antimicrobial activity and sealing capacity.

From this perspective, chlorhexidine-hexametaphosphate nanoparticles in AH Plus, MTA Fillapex, and Pulp Canal Sealer resulted in increased antimicrobial activity across all evaluated materials; even though a reduction in flow and an increase in solubility were observed after 24 h, there was no compromise in radiopacity, indicating potential for antimicrobial performance with limited impact on their physicochemical properties [75].

Epoxy resin-based endodontic sealers (ERBS) are considered the gold standard in endodontic treatment due to their dimensional stability, low solubility, adequate flowability, and sealing ability [178]. These properties are associated with the formation of a highly cross-linked polymeric network during setting, resulting from the reaction between epoxide monomers and polyamine hardeners, which limits water penetration and material degradation [179,180].

To further enhance their properties, different nanoparticles can be incorporated. The studies show considerable heterogeneity regarding the type, size, and concentration of nanoparticles used, reflecting diverse strategies aimed at improving antimicrobial and bioactive properties.

Studies on nanoparticle incorporation into epoxy resin-based endodontic sealers report diverse compositions, including silver-based systems, quaternary ammonium compounds, chitosan, magnesium hydroxide, bioactive glass, hydroxyapatite, and chlorhexidine-derived nanoparticles [75,77,78,79,181,182,183,184]. These approaches generally aim to enhance antimicrobial and bioactive properties without compromising physicochemical performance [75]. However, marked heterogeneity in nanoparticle type, size, and concentration limits direct comparisons and precludes standardized protocols [185]. Although lower concentrations tend to better balance stability and performance, improvements in biological properties are not consistently achieved without affecting parameters such as solubility and flowability, underscoring the need to balance bioactivity with material stability [80].

### 7.4. Nanotechnology in Regenerative Endodontics

Regarding endodontic revascularization, the main goal is to re-establish a biological microenvironment capable of supporting the formation of pulp-like tissue within previously necrotic root canals, while also promoting continued root development [186]. In experimental models of infected immature teeth, mesoporous silica nanoparticle (MSN) scaffolds, with or without BMP-2 incorporation, were able to increase root length and thickness, as well as stimulate vital tissue formation and hard tissue deposition. Superior outcomes were observed when combined with BMP-2, which also reduced the inflammatory response [86].

In parallel, the association of gold nanoparticles (AuNPs) with advanced platelet-rich fibrin (A-PRF) demonstrated significant antimicrobial activity against Enterococcus faecalis, in addition to the potential for sustained growth factor release, contributing to canal sterility maintenance and supporting successful revascularization. Collectively, these findings suggest that nanoparticles may represent a promising strategy for regenerative endodontic therapy [87].

## 8. Biological Safety and Toxicity

Toxicity is not an isolated issue but represents a central translational limitation for nanoparticle-based systems. Although nanomaterials exhibit favorable properties for dental applications, biosafety and toxicity remain important limitations to their clinical use, as adverse biological responses may vary according to nanoparticle composition and physicochemical characteristics.

Metallic nanoparticles such as Ag, TiO_2_, ZnO, CuO, and Au have been associated with oxidative stress, inflammation, apoptosis, DNA damage, and mitochondrial dysfunction [187,188,189,190,191]. In addition to potential systemic toxicity, exposure may occur through ingestion, material wear, and aerosol generation during clinical procedures [192,193], and released particles may reach adjacent tissues and cross biological barriers, including the blood–brain barrier [193,194,195].

These concerns are especially relevant for restorative materials exposed to the oral environment, where dissolution, wear, erosion, and pH fluctuations may promote degradation and release of metallic ions [196,197,198,199]. The inherent instability of nanomaterials in fluctuating oral pH makes their clinical performance unpredictable. This instability can trigger the unintended release of metallic ions. Once released, these ions enter the systemic circulation and may be internalized by cells [200]. Following cellular uptake, these ions may undergo degradation and exocytosis but can also induce cytotoxic, genotoxic, and inflammatory responses [196,201].

However, biological responses are not uniform across nanosystems. Some materials, including AgNPs in endodontic irrigants and Zr nanoparticles in cements, show low cytotoxicity and favorable outcomes [197,198,199]. Polymer-based systems such as PCL, PLGA, PEG-conjugated polymers, and liposomes generally exhibit good biocompatibility and biodegradability, with established biomedical applications [202,203,204,205,206]. Chitosan nanoparticles are also widely considered biocompatible, although concentration-, size-, and exposure-dependent cytotoxicity has been reported [207].

The variability of toxicological outcomes is further illustrated by hydroxyapatite nanoparticles (HAp NPs), whose biological effects remain controversial. While Somez et al. observed dose-dependent toxicity in rat liver cells, including increased cytotoxicity, micronucleated hepatocytes, and elevated 8-OH-dG levels at high concentrations (300–1000 μg cm^−2^) [208], Remya et al. 2014, reported no toxicity in bone marrow mesenchymal stem cells at concentrations up to 800 μg/mL [209]. Moreover, oral administration of HAp NPs to Wistar rats for one year did not induce chronic toxicity [210].

Overall, the safety profile of nanomaterials cannot be generalized across classes [143]. Their biological effects depend on composition, concentration, particle size, exposure time, and release kinetics, while methodological heterogeneity limits standardized safety thresholds [211]. Therefore, clinical translation requires long-term studies, standardized protocols, and comprehensive toxicological evaluation to better define their risk–benefit balance [171,212,213].

## 9. Conclusions

Nanotechnology has significantly expanded therapeutic possibilities in restorative dentistry and endodontics, with a wide range of nanomaterials applied to caries prevention, remineralization, adhesive modification, development of functional restorative materials, irrigants, intracanal medicaments, sealers, and regenerative strategies. However, the available evidence is predominantly based on in vitro studies, with fewer in vivo investigations and limited clinical validation. In this context, systems such as silver nanoparticles, nano-hydroxyapatite, calcium/phosphate-based bioactive materials, and nanomaterials incorporated into adhesives and restorative materials appear to be the closest to clinical application, particularly due to their consistent antimicrobial, remineralizing, and material-enhancing effects.

In contrast, more complex approaches, including multifunctional regenerative systems, stimulus-responsive platforms, and advanced controlled-release technologies, remain largely experimental and still require validation in preclinical models and well-designed clinical studies. Thus, despite the strong translational potential of nanomaterials, their clinical incorporation is currently limited not only by the predominance of laboratory-based evidence but, fundamentally, also by critical concerns regarding long-term biosafety. Future research must prioritize standardized toxicological assessments and longitudinal clinical trials to establish a clear risk-benefit profile, ensuring the safe and predictable integration of nanotechnology into routine dental practice.

## Figures and Tables

**Table 1:** Nanoparticles applied in restorative dentistry.

Category	Study Type	Nanoparticles/Systems	Main Applications	References
Nanoparticles Applied to Prevention	In situ/in vivo experimental study	Casein Phosphopeptide-Amorphous Calcium Phosphate (CPP-ACP) nanocomplexes	Calcium/phosphate delivery; enamel remineralization	[24]
	Randomized controlled trial	Casein Phosphopeptide-Amorphous Calcium Phosphate (CPP-ACP) nanocomplexes	Early childhood caries prevention; remineralization	[25]
	Randomized double-blind clinical trial	Silver nanoparticles associated with xylitol	Biofilm control; remineralization of orthodontic white spot lesions	[26]
	In vitro	Nano-calcium carbonate (nano-caco_3_)	Enamel remineralization; calcium supply	[27]
	In vitro	Calcium glycerophosphate (β-cagp)	Biofilm modulation; buffering; mineral replenishment	[28]
	In vitro	Calcium glycerophosphate (β-cagp)	Activity against cariogenic biofilms (*S. mutans* and *C. albicans*)	[29]
	In vitro	Arginine and Calcium glycerophosphate (β-cagp)	Biofilm fluid modulation; ion concentration control	[30]
	In vitro	Nanosized β-calcium glycerophosphate	Biomineralization; remineralizing potential in toothpastes	[31]
	In vitro	Silver nanoparticles (agnps)	Recurrent caries prevention in xerostomic patients	[32]
	In vitro	Nano-hydroxyapatite (nhap)	Reduction of dentin hypersensitivity	[33]
	In vitro	Nano-hydroxyapatite (nhap)	Enamel surface remineralization via toothpaste	[34]
	In vitro	Nano-fluorapatite (nfa)	Hypersensitivity treatment; controlled fluoride release	[35]
	In vitro	Nano-fluorapatite (nfa)	Long-term fluoride ion release assessment	[36]
	In vitro	Nano-fluorapatite (nfa)	Development of fluoride release systems	[37]
Nanoparticles in Adhesives	In vitro	Silver nanoparticles (agnps)	Antibacterial activity; biofilm inhibition in adhesives	[38]
	In vitro	Self-assembled oligopeptide amphiphile	Biomimetic enamel mineralization	[39]
	In vitro	Amorphous calcium phosphate (NACP)	Dentin remineralization under biofilm challenge	[40]
	In vitro	Bioactive glass nanoparticles (bgns)	Mineral deposition; antibacterial; MMP inhibition	[41]
	In vitro	Bioactive glass nanoparticles	Biomimetic remineralization of human dental enamel via bioactive glasses	[42]
	In vitro	Nano-hydroxyapatite (nhap)	Reduction of collagen degradation in demineralized dentin	[43]
	In vitro	Zinc nanoparticles (znnps)	MMP inhibition; Bioactivity; preservation of adhesive bond integrity	[44]
	In vitro	Gold nanoparticles (aunps)	Matrix metalloproteinase (MMP) inhibition; biocompatibility	[45]
	In vitro	Mesoporous silica nanoparticles (msns)	Physical occlusion of dentinal tubules	[46]
	In vitro	Aluminosilicate nanotubes (alnts)	Dentin sealing; improvement of bonding properties	[47]
	In vitro	Copper nanoparticles / cui	Antimicrobial activity; resin-dentin interface durability	[48]
	In vitro	Β-tricalcium phosphate nanoparticles (β-TCP)	Increase in push-out bond strength in weakened roots	[49]
Functional Nanoparticles for Restorative	In vitro	Cross-linked quaternized polyethyleneimine (QPEI) nanoparticles	Contact-killing antibacterial effect against *S. mutans*	[50]
	In vivo	Cross-linked quaternized polyethyleneimine (QPEI) nanoparticles	Biofilm stress induction; selective cell death in vivo	[51]
	In vitro	Silica–sic nanostructures (CHX-functionalized)	Mechanical reinforcement; remineralization; antibacterial action	[52]
	In vitro	Titanium dioxide (tio_2_-nps)	Bacterial response modulation; mechanical reinforcement	[53]
	In vitro	Hybrid Ag/zno and Ag/cuo nps	Synergistic antimicrobial activity with strain-specific sensitivity profiles	[54]
	In vitro	Silver nanoparticles (agnps)	Synergistic antimicrobial efficacy and biofilm growth inhibition	[55]
	In vitro	Silver nanoparticles (agnps)	Antimicrobial; ion release; anti-biofilm (*K. pneumoniae*)	[56]
	In vitro	Silver nanoparticles (agnps)	Did not show cytotoxic effects	[57]
	In vitro	Zinc oxide (zno-nps)	Antimicrobial; biofilm reduction (*P. aeruginosa*); durability	[58]
	In vitro	Nickel oxide nanoparticle	Compromise structural integrity and bacterial adhesion in *Escherichia coli*	[59]
	In vitro	Titanium dioxide nanoparticle	Causes mutations through oxidative stress in *S. typhimurium*	[60]
	In vitro	Copper nanoparticles	Mechanical properties; drug release modeling; antimicrobial	[61]
	In vitro	Superparamagnetic Iron Oxide Nanoparticles (Spions)	Magnetic guidance to enhance adhesive infiltration and sealing performance under simulated pulpal pressure	[62]
	In vitro	Superparamagnetic Iron Oxide Nanoparticles (Spions)	Multifunctional reinforcement to counteract adhesive interfacial breakdown and improve long-term bond durability	[63]
	In vitro	Gold nanoparticles (aunps)	Mechanical reinforcement; antibacterial features in adhesives	[64]
Materials Auxiliary Function Nanoparticles	In vitro	Chitosan nanoparticles	Antimicrobial; anti-erosive; drug delivery; adhesion improvement	[65]
	In vitro	Chitosan hydrogels (with amelogenin)	Minimization of enamel loss after erosive challenge	[66]
	In vitro	Propolis/nystatin/β-carotene nanocomposites	Antibacterial activity; inhibition of enamel demineralization	[67]
	In vitro	Modified glass ionomer with chitosan nanoparticles	Modification of mechanical and biological properties in GIC	[68]

**Table 2:** Main nanoparticle systems investigated for endodontic applications.

Category	Study Type	Nanoparticle/System	Main Applications	References
Irrigating solutions and intracanal medicaments	In vitro	Silver nanoparticles (AgNPs)	Residual antibacterial effects against *E. faecalis*, biofilm disruption, dentinal tubule penetration	[69]
	In vitro	Chitosan nanoparticles (CSNPs)	Efficacy against *E. faecalis* and *C. albicans* biofilms	[70]
	In vitro	Cerium nitrate + dextran system	Biomimetic mineralization; antibacterial activity; enhancement of bioceramic clinical applications	[71]
	Randomized Controlled Trial	Jasminum-based Nano-reinforced Calcium Hydroxide	aimed at reducing postoperative pain and enhancing antimicrobial activity through nanoparticles.	[72]
	In vitro	Polydopamine nanoparticles (PDA)	NIR-activated photothermal biofilm eradication	[73]
	In vitro	Laser-activated nanoparticle systems	Enhanced antimicrobial efficacy, dentinal tubule disinfection	[74]
Endodontic sealers and filling materials	In vitro	Chlorhexidine-hexametaphosphate nanoparticles	Antibacterial and physicochemical reinforcement of sealers	[75]
	In vitro	Quaternary ammonium nanoparticles	Antibacterial activity, biofilm control	[76]
	In vitro	Silver nanoparticles/silver vanadate systems	Improvement of sealer physical and antibacterial properties	[77]
	In vitro	Bioactive glass nanoparticles	Bioactivity evaluation; physical property enhancement	[78]
	In vitro	Hydroxyapatite nanoparticles	Sealing enhancement; remineralization potential	[78]
	In vitro	Magnesium hydroxide nanoparticles	pH modulation; antimicrobial activity in sealers	[79]
	In vitro	Chitosan nanoparticles	Flowability, solubility, and setting time modification	[79]
	In vitro	Chlorhexidine nanoparticles (CHX-HMP)	Controlled ion release; pH elevation in cements	[80]
	In vitro	Copper nanoparticles	Particle size-dependent ion release; antimicrobial	[80]
	In vitro	Zinc oxide nanoparticles (ZnO)	Biofilm reduction; antimicrobial activity in endodontic cements	[80]
Regenerative endodontic formulations	In vitro	Natural mineralized scaffolds	Dentinogenic potential of stem cells	[81]
	In vitro	Cerium oxide nanoparticles (CNPs)	Inflammation regulation; pulp regeneration support	[82]
	In vitro	DMP1-based systems	Induction of odontogenic differentiation	[82]
	In vivo	Hydroxyapatite nanoparticles (nano-HAP)	Pulp-capping agent for mineralized tissue formation	[83]
	Prospective clinical study	Magnetic nanoparticles (MNPs)	Hard tissue barrier formation in primary molar pulpotomy	[84]
	In vitro	Mesoporous bioactive glass nanoparticles (MBGNs)	Bioactivity; apatite formation in doped glass nanoparticles	[85]
	In vivo	Mesoporous silica nanoparticles (MSNs)	Root maturation and tissue regeneration in immature teeth	[86]
	In vitro	Gold nanoparticles + A-PRF	Revascularization of necrotic immature permanent teeth	[87]

## Data Availability

No new data were created or analyzed in this study. Data sharing is not applicable to this article.

## Abbreviations

The following abbreviations are used in this manuscript:

Abbreviation	Full Term
AgNPs	Silver nanoparticles
ZnO	Zinc oxide
nHAp/nHAP	Nano-hydroxyapatite
NPs	Nanoparticles
AlNTs/Al-NTs	Aluminosilicate nanotubes
nc-CHA	Carbonated nanohydroxyapatite
Zn-CHA	Zinc-doped carbonated hydroxyapatite
nFA	Nano-fluorapatite
n-CaF_2_	Nano-calcium fluoride
nano-CaCO_3_/NC	Nano-calcium carbonate
CPP-ACP	Casein phosphopeptide–amorphous calcium phosphate
CHX	Chlorhexidine
NACP	Amorphous calcium phosphate nanoparticles
BGNs	Bioactive glass nanoparticles
ZnNPs	Zinc nanoparticles
AuNPs	Gold nanoparticles
CPNs	Colloidal platinum nanoparticles
MSNs	Mesoporous silica nanoparticles
RNGs	Reactive nanogels
CuI	Copper iodide
β-TCP	Beta-tricalcium phosphate
TiO_2_-NPs	Titanium dioxide nanoparticles
SPIONs	Superparamagnetic iron oxide nanoparticles
QPEI	Quaternized polyethyleneimine
CHX-HMP	Chlorhexidine hexametaphosphate
CSNPs	Chitosan nanoparticles
PDA	Polydopamine nanoparticles
NIR	Near-infrared
ERBS	Epoxy resin-based sealers
CNPs	Cerium oxide nanoparticles
DMP1	Dentin matrix protein 1
FGF-2	Fibroblast growth factor 2
MNPs	Magnetic nanoparticles
MBGNs	Mesoporous bioactive glass nanoparticles
BMP-2	Bone morphogenetic protein 2
A-PRF	Advanced platelet-rich fibrin
ROS	Reactive oxygen species
Ca(OH)_2_	Calcium hydroxide
Ca^2+^	Calcium ion
PO_4_^2−^	Phosphate ion
NaF	Sodium fluoride
MMPs	Matrix metalloproteinases
hDPSCs	Human dental pulp stem cells
